# Evaluation of Bioprocess-Based Technique for Iron and Zinc Fortification in Red Rice Genotypes

**DOI:** 10.3390/foods14183162

**Published:** 2025-09-11

**Authors:** Sai Sruthi Shree Kavitha Kumaravel, Nagarajan Srividya

**Affiliations:** Department of Food and Nutritional Sciences, Sri Sathya Sai Institute of Higher Learning, Anantapur 515001, India; saisruthishreekk@sssihl.edu.in

**Keywords:** heritage rice, Chennangi, Karungkuruvai, iron fortification, zinc fortification, double fortification, germination, bioaccessibility, cooking quality, EDI

## Abstract

Iron (Fe) and zinc (Zn) deficiencies, globally prevalent nutritional disorders, underscore the need for effective fortification strategies in staple foods like rice. This study evaluates a bioprocess-based technique for single (SF) and double fortification (DF) of two heritage red rice genotypes (Chennangi—CH, Karungkuruvai—KK) to enhance mineral content and bioavailability. Whole rice grains were germinated in sodium iron EDTA and zinc chloride solutions (SF: 50 and 100 mg/L Fe/Zn; DF: Fe + Zn at a 2:1 ratio). Mineral quantification via microwave plasma atomic emission spectrometry (MPAES) revealed that SF significantly increased fortified mineral content but reduced accessibility of the non-fortified mineral. In contrast, DF substantially enhanced both Fe (2-fold) and Zn (7-fold) content while improving bioaccessibility (Fe: 2–2.5x; Zn: 3–7x), supported by reduced phytate levels. Both genotypes exhibited high Zn accumulation and retention. Cooked DF rice has good sensory acceptability and improved cooking characteristics. At daily consumption levels of 30–150 g, DF rice could meet 16–70% of Fe and nearly 100% Zn Recommended Dietary Intake (RDI) across age groups. This simple, scalable bioprocessing method effectively enhances Fe and Zn bioavailability in wholegrains, offering a promising solution to combat micronutrient deficiencies through dietary staples, contributing to Sustainable Development Goals (SDG 2 and 3) by promoting accessible nutrition for healthier populations.

## 1. Introduction

Micronutrient deficiencies, particularly iron and zinc deficiencies, remain a significant global public health challenge. According to the Global Nutrition Report 2021, over 1.6 billion people worldwide suffer from iron deficiency anemia, while zinc deficiency affects approximately 17% of the global population, leading to increased morbidity and mortality, especially among children and women of reproductive age [1]. Despite concerted efforts by governments and researchers, including large-scale supplementation and fortification programs in various countries, the prevalence of these deficiencies remains alarmingly high. Recent surveys in India indicate that about 57% of women and 67% of children under five suffer from anemia [2,3,4]. Zinc deficiency is also a widespread concern, affecting roughly 17–31% of children and adolescents nationwide [2]. On a global scale, zinc deficiency impacts an estimated 17% of the population, with mild to moderate deficiency common worldwide and contributing to significant child illness and mortality, especially in low- and middle-income countries [5,6,7]. Additionally, recent estimates suggest that approximately 40% of Indian children aged 12–59 months are anemic, with nearly 30% of these cases linked to micronutrient deficiencies, primarily iron [8].

Fortification of food is a cost-effective strategy to improve the nutrition status of populations [9]. Staple foods have been widely recognized as effective vehicles for micronutrient fortification due to their regular and widespread consumption across diverse populations [10,11]. Cereals such as rice, wheat, and maize are particularly suitable because they form the dietary backbone in many countries, ensuring a broad reach and impact of fortification programs [12,13].

India’s rice fortification uses mainly extrusion technology, producing fortified rice kernels (FRKs) that are blended with regular rice. These are centrally produced and distributed through government programs like the Public Distribution System and Mid-Day Meals, apart from coating and dusting methods [14,15]. Biofortification through breeding and genomic approaches has produced several zinc- and protein-rich rice varieties, such as CR Dhan 310 and Zinco Rice-MS [16]. Parboiling fortification, another alternative method, incorporates micronutrients into rice during the soaking and steaming process [17].

However, the current fortification methods face certain challenges. FRKs are centrally manufactured and distributed with high investment costs. Coating and dusting methods face issues like nutrient loss and sensory changes (color and flavor). Biofortified crops are dependent on agricultural preferences and seed availability by farmers, and limited consumer acceptance of genetically modified crops. The Parboiling method, though promising, faces challenges related to nutrient leaching. These limitations necessitate complementary strategies and continuous innovation to optimize rice fortification’s impact in India.

Pigmented rice varieties, such as red, black, and purple rice, remain underexplored as fortification vehicles despite their inherent advantages. These varieties are culturally accepted and widely consumed in various regional cuisines, often prepared in diverse culinary forms. Their natural pigmentation, due to anthocyanins and other polyphenols, provides a unique advantage: fortification is less likely to cause visible changes in color or taste, thereby enhancing consumer acceptance [18]. Moreover, pigmented rice varieties are nutritionally superior to polished white rice, offering higher levels of protein, dietary fiber, antioxidants, and essential micronutrients such as iron and zinc [19]. Emerging evidence also suggests that pigmented rice may offer additional benefits for individuals with chronic metabolic conditions, including diabetes, due to its lower glycemic index and higher fiber content [20,21].

Pigmented and whole-grain rice varieties also face specific challenges in terms of consumer acceptance. The intact bran layer contributes to longer soaking and cooking times, which may be less appealing to modern consumers. In addition, the bran contains phytates and other antinutritional compounds that bind to minerals such as iron and zinc, thereby lowering their bioavailability [22]. This necessitates innovative processing techniques to enhance mineral absorption.

In response to these challenges, bioprocessing methods, such as germination and fermentation, have demonstrated significant potential to improve the nutritional quality and functional properties of whole grains [23,24]. Germination, in particular, activates endogenous enzymes that degrade phytates, increase bioactive compounds, and improve protein digestibility. Germinated brown rice has been extensively studied, showing enhanced antioxidant capacity, increased mineral bioavailability, and improved sensory qualities compared to non-germinated counterparts [25,26].

In the present study, we aimed to evaluate the impact of bioprocess-based single and double fortification methods on the total and bioaccessible content of iron and zinc, and study the mineral uptake behavior and matrix interactions in two heritage red rice varieties. We also hypothesized that the chosen bioprocess method for fortification (germination) may positively influence the cooking and sensory attributes of the experimental samples, improving sensory acceptability and consumer convenience. To the best of our knowledge, germination-based mineral fortification of red rice genotypes has not been systematically reported in the literature, particularly with reference to Fe and Zn and their interaction.

## 2. Materials and Methods

**Figure 1:**
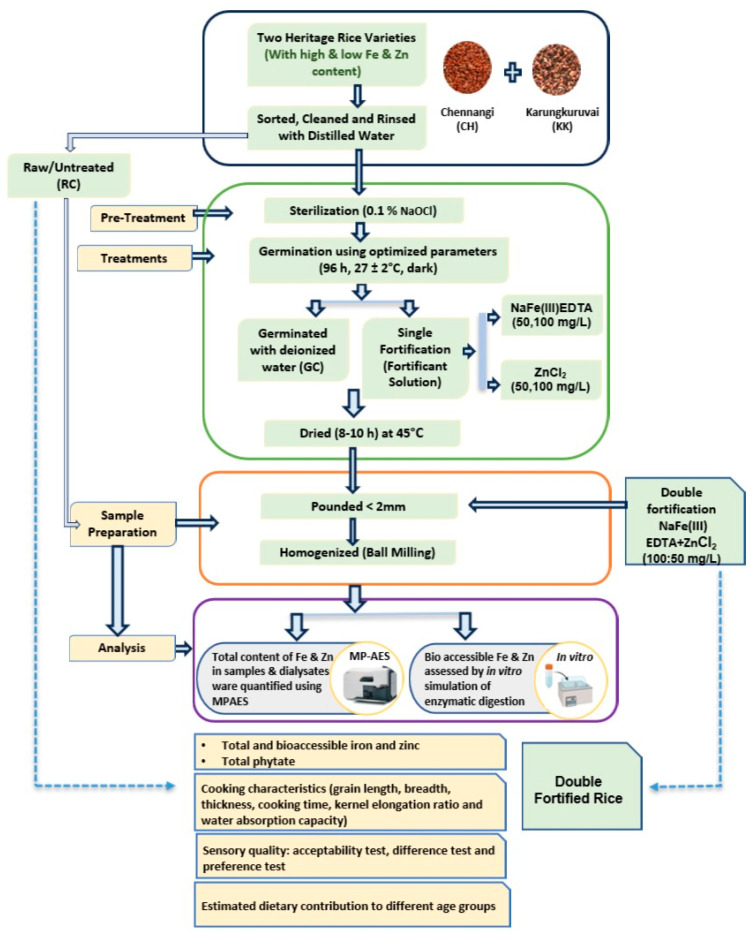
Experimental Design.

The experimental design depicting the overall workflow of the study, including sample preparation, germination, fortification, and analytical evaluations, is illustrated in Figure 1.

### 2.1. Rice Samples and Chemicals

Essential mineral quantification was performed on twenty-four pigmented heritage rice varieties in our laboratory (unpublished data). From these, two red-pigmented Indian heritage rice varieties with varying content of iron (Fe) and zinc (Zn) were selected for the current study: Chennangi (CH), which has higher total Fe and Zn content, and Karungkuruvai (KK), which has lower levels of these minerals. CH was obtained from Dharani Farms through Timbaktu organization in the state of Andhra Pradesh, and KK was directly procured from the farmers in Tamil Nadu state, India. The whole grains were then stored at −20 °C until further use. All chemicals used in the study were of analytical grade obtained from Merck (Darmstadt, Germany).

### 2.2. Bioprocess-Based Single and Double Fortification

The selected HRVs were first sorted to remove damaged grains and rinsed thoroughly with distilled water. The raw, untreated samples were used as the basic control samples and designated as RC.

Germination was selected as the bioprocess method. The whole rice grains were germinated using the method given by [25] with slight modifications using deionized water without the addition of any fortificants. This was taken as the germination control (GC).

In the first phase, single fortification trials were carried out. For single fortification (SF) with iron, HRVs were treated with Fe(III)EDTA solution (50 and 100 mg/L). Fe(III)EDTA was selected as the iron fortificant for its stability, bioavailability, and better performance in phytate-rich foods, as the EDTA chelation reduces the inhibitory effect of phytates on iron absorption [27]. Single fortification with zinc was carried out using ZnCl_2_ (50 and 100 mg/L) solution prepared in deionised water. ZnCl_2_ was chosen as the zinc fortificant owing to its high solubility and reported efficient absorption into grain matrices [28]. The reported concentrations denote the levels of fortificant salts expressed in mg/L of solution.

In the second phase, double fortification (DF) with iron and zinc was carried out using a mixed fortificant solution with Fe and Zn at concentrations of 100 and 50 mg/L, respectively. The Fe:Zn ratio was selected based on Phase 1 trials, taking into account the uptake capacity of HRVs for each mineral and the need to minimize competitive inhibition.

For germination and bioprocess-based fortification studies, the above-mentioned whole rice grain samples were placed in sterilized germination trays and soaked completely in the respective fortificant solutions with a grain (weight) to solution (volume) ratio of 1:3. The trays were then placed in an incubator at 27 ± 2 °C in the dark. The solution was changed every 6–8 h, and closely monitored for any fungal growth or off odor. After 96 h, the germinated grains were removed, rinsed twice with deionized water, and dried overnight at 45 °C using a food dehydrator (Hendi make, China).

### 2.3. Sample Preparation for Analyses

The dried samples were ground using an agate mortar and pestle to obtain a size reduction to ˂2 mm. The ground samples were then homogenized using a planetary ball mill (Pulverisette, Premium line, Fritsch, Germany). All the samples were homogenized under standardized parameters such as ball-to-sample ratio (4.5:1), rpm (700), and total run time (10 min). Care was taken to use agate sample holders as well as balls to avoid any kind of metal contamination. 

### 2.4. Quantification of Total Iron and Zinc

Iron and zinc were quantified using the MP-AES method adapted from [29]. Homogenized samples (0.5 g) were digested with 6 mL concentrated nitric acid. The tubes were placed in a water bath at 100 °C for 30 min and subsequently in an ultrasonicator (Branson Bransonic® Digital Bath 5800, St. Louis, MO, USA) at 45 °C for 30 min. The volume of the digests was then made up to 100 mL and filtered. An aliquot of 10 mL was taken for analysis. The final solution used for measurement had <4% TDS content. Quantification was performed using the Microwave Plasma Atomic Emission Spectrophotometer (Agilent 4200, Agilent Technologies, Santa Clara, CA, USA) equipped with a hermetically sealed, UV-sensitive, back-thinned solid state CCD detector and an autosampler. Triplicate measurements were made, and both minerals were quantified based on the standard curve obtained for the respective mineral from the ICP multi-element standard VIII (Merck). A certified Reference Material (CRM) No. 10 d Rice flour-unpolished obtained from the National Institute of Environmental Studies (NIES), Japan, was also used to validate the accuracy of the method.

### 2.5. Determination of In Vitro Iron and Zinc Bioavailability

The in vitro bioavailability and dialysability of iron were assessed following the method described by Luten et al. [30] with minor modifications to accommodate the sample characteristics. Homogenized sample (0.5 g) was suspended in 20 mL of distilled water and subjected to sonication for 2 min using a probe sonicator (1/8” probe, Branson Sonifiers SFX 150, St. Louis, MO, USA). For the gastric digestion phase, 938 µL of pepsin solution was added to the homogenate, and the total volume was adjusted to 25 mL. The mixture was incubated in a shaking water bath at 37 °C for 2 h. Following incubation, the pepsin digest was cooled on ice. To determine titratable acidity, 10 mL of the pepsin digest was combined with 2.5 mL of a pancreatin-bile extract mixture and titrated with 0.5 N NaOH to pH 7.5. The volume of NaOH required was recorded as ‘X’. Subsequently, 15 mL of the pepsin digest was transferred to prepared dialysis tubing containing 12.5 mL of distilled water and X mL of 0.5 M NaHCO_3_. The assembly was incubated in a shaking water bath at 37 °C until the pH reached 5. At this point, 2.5 mL of the pancreatin-bile extract mixture was added, and incubation continued for an additional 2 h. Upon completion of the incubation, the dialysis tubing was removed and rinsed thoroughly with distilled water. The dialysate was collected, weighed, and analyzed for dialyzable mineral content using MP-AES (Agilent 4200, Agilent Technologies, Santa Clara, CA, USA).

### 2.6. Estimation of Total Phytate

Total phytate content was determined according to the method described by [31], with minor modifications. Homogenized sample (35 mg) was placed in an Eppendorf tube, to which 1 mL of 0.4 N HCl was added. The mixture was agitated on a platform shaker at 200 rpm for 3.5 h at room temperature. Following extraction, the tubes were centrifuged at 3900× *g* for 15 min, and the resulting supernatant was collected for further analysis. For the colorimetric assay, 35 µL of the sample extract was transferred to an Eppendorf tube, followed by the addition of 35 µL distilled water and 140 µL of 0.02% (*w*/*v*) ammonium iron (III) sulfate in 0.2 N HCl. The mixture was heated in a water bath at 99 °C for 30 min, then cooled at 4 °C for 15 min, and subsequently incubated at room temperature for 20 min. The tubes were then centrifuged at 3900× *g* for 30 min at 24 °C. An aliquot of 85 µL from each supernatant was mixed with 120 µL of 1% (*w*/*v*) 2,2′-bipyridine and 1% (*v*/*v*) thioglycolic acid. Absorbance was measured immediately at 519 nm using a multimode reader (Varioskan LUX, Thermo Fisher Scientific, Waltham, MA, USA). Phytic acid (Sigma-Aldrich, St. Louis, MO, USA) was used as the standard, with a calibration curve prepared using concentrations ranging from 5 to 25 mg/mL.

### 2.7. Grain and Cooking Characteristics

The grain and cooking characteristics of the raw/unprocessed (RC) and double-fortified (DF) HRVs for both uncooked and cooked forms were evaluated using the methods described by Thavamurugan et al. [32].

Length and breadth: The length and width of ten intact, undamaged grain kernels from each cultivar were measured using a vernier caliper and reported in millimeters. The length-to-breadth (L/B) and breadth-to-thickness (B/T) ratios and normalized grain weight were subsequently calculated.

Cooking time: The cooking time was determined by placing 2 g of rice in a test tube with 20 mL of deionized water. The rice grains were then heated at 90 °C till they formed a cohesive mass without a visible white core.

Water uptake ratio: The weight of cooked rice was divided by the weight of raw rice to determine the water absorption ratio.

Ratio of kernel elongation: The kernel elongation ratio (KER), indicative of rice’s cooking quality, was determined by comparing the average length of ten uncooked grains to that of ten cooked grains of the samples. KER was calculated using the following formula

KER = (XL − YL)/YL

where XL is the mean length of ten cooked grains and YL is the mean length of ten uncooked grains.

### 2.8. Determination of Sensory Quality—Acceptability Test, Preference Test, and Difference Test

Sensory acceptability studies were carried out for the raw/unfortified (RC) and double-fortified HRVs by preparing a standardized tomato bath recipe. The overall acceptability was evaluated along with other sensory attributes such as appearance, color, taste, flavor, and texture, using a 9-point hedonic scale by 24 adult panel members (untrained).

DF-HRVs were subjected to two additional tests—the triangle test and preference test.

The triangle test is a sensory discrimination method in which panelists (24 semi-trained) are presented with three coded samples, two of which are identical, and asked to identify the odd sample, thereby assessing whether a perceptible difference exists between products [33]. The preference test, on the other hand, asks panelists to indicate which of two samples they prefer, providing direct information about consumer liking [34]. 

The sensory assessment of the samples was conducted in accordance with international best practices for sensory evaluation studies, following guidelines provided by the Institute of Food Science and Technology, London. The study adhered to standard ethical guidelines, ensuring voluntary participation, informed consent, and strict confidentiality of all collected data.

### 2.9. Estimation of Dietary Mineral Contribution from Double-Fortified Pigmented Rice Varieties

Determination of dietary mineral contribution acts as a potent tool in evaluating the capability of any food in meeting the mineral requirements of the population [35]. First, the estimated daily intake (EDI) of Fe and Zn from DF pigmented rice varieties was calculated based on the content of these minerals, considering an intake of 25, 50, 75, 100, 125, 150, and 175 g of reference amount consumed (RAC).

$$\mathrm{EDI}\;\left(\mathrm{mg}/\mathrm{day}\right)=\frac{\mathrm{Nutrient}\;\mathrm{Content}\;\left(\mathrm{mg}/100\;g\right)}{100}\times \mathrm{RAC}\;\left(g\right)$$

where EDI = Estimated daily intake; RAC = Reference amount consumed.

Then, based on the EDI, the dietary iron and zinc contribution for different age groups was computed against the RDI (U.S. Food and Drug Administration) as given below:

$$\mathrm{Dietary}\;\mathrm{Mineral}\;\mathrm{contribution}\;\left(\%\right)=\frac{\mathrm{EDI}}{\mathrm{RDI}}\times 100$$

where RDI = Reference daily intake.

RDI values are based on the U.S. Food and Drug Administration (FDA) Reference Daily Intakes (RDI) for adults and children.

### 2.10. Statistical Analysis

Results were expressed as means of three independent values along with standard deviations. Analysis of Variance test (ANOVA) followed by post hoc Tukey HSD test at *p* ≤ 0.05 was performed to understand the differences in the analyzed mineral parameters, phytic acid content, and cooking characteristics among the experimental samples. The sensory analysis results were expressed as the mean values obtained from the participants. Analysis was performed using IBM SPSS Statistics 21, IBM, Armonk, NY, USA.

## 3. Results

### 3.1. Effect of Germination on Total and Bioaccessible Iron and Zinc Content

**Figure 2:**
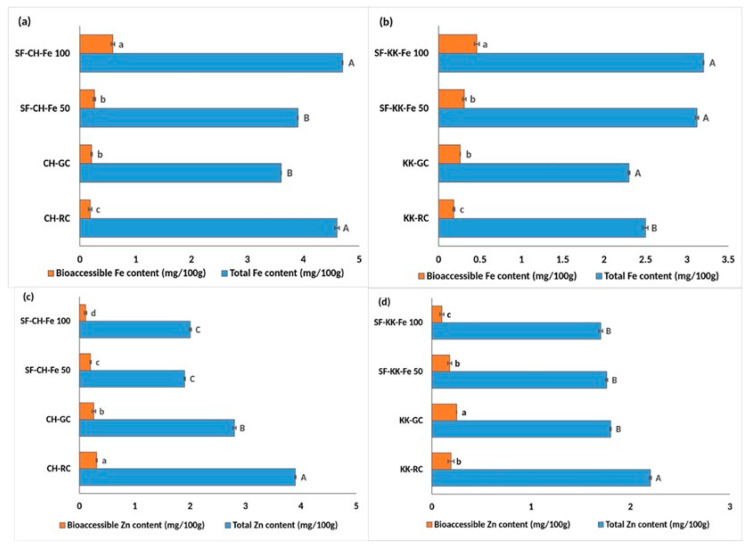
Effect of single fortification with NaFeEDTA on the total and bioaccessible iron and zinc content in raw, germinated, and fortified Chennangi and Karungkuruvai rice samples. (**a**) Total and bioaccessible iron content in Chennangi samples; (**b**) Total and bioaccessible iron content in Karungkuruvai samples; (**c**) Total and bioaccessible zinc content in Chennangi Samples; (**d**) Total and bioaccessible zinc content in Karugkuruvai samples. Different lowercase letters (a–c) indicate significant differences (*p* < 0.05) in bioaccessible content among treatments within the CH and KK sample sets. Different uppercase letters (A–C) indicate significant differences (*p* < 0.05) in total content among treatments within the CH and KK sample sets. Data are presented as mean ± SD of three independent determinations; different alphabets indicate significant differences at *p* ≤ 0.05. (Note: CH—Chennangi, KK—Karungkuruvai; the suffixes R—raw/unprocessed, G—control/germinated, SF—Fe (50/100)—iron fortified counterparts of different concentrations expressed in mg/L).

Germination showed a varied influence on iron (Fe) and zinc (Zn) levels in both varieties in comparison with the raw untreated samples. In CH, the total Fe content (Figure 2a) significantly reduced (at *p* < 0.05) from 4.6 in RC to 3.6 mg/100 g in GC; however, the amount of bioaccessible Fe increased significantly (at *p* < 0.05) from 0.185 to 0.21 mg/100 g (Figure 2a), indicating improved solubility and availability.

In KK, both the total Fe and its bioaccessible fraction decreased from 2.5 to 2.3 mg/100 g (Figure 2c) and from 0.18 to 0.24 mg/100 g (Figure 2c), respectively, in KK-RC and KK-GC. In contrast, zinc responded differently. Germination led to a significant (at *p* < 0.05) reduction in total Zn in both CH (3.9 to 2.8 mg/100 g) and KK (2.2 to 1.8 mg/100 g). Despite this decline, the bioaccessible Zn fraction significantly improved in KK (0.19 to 0.25 mg/100 g), whereas in CH it decreased significantly from 0.31 to 0.26 mg/100 g.

### 3.2. Single Fortification with Iron—Effect on Total and Bioaccessible Iron and Zinc Content

The respective germinated samples (GC) were considered as the controls for the fortified samples, and their values are compared in the results.

Bioprocess-based single fortification with iron fortificant salt markedly enhanced the nutritional quality of Fe in both CH and KK varieties (Figure 2a and Figure 2b, respectively). In CH, total iron content increased from 3.6 mg/100 g in CH-GC to 3.9 mg/100 g in SF-CH-Fe 50, and to a significantly (*p* < 0.05) higher value of 4.7 mg/100 g in SF-CH-Fe 100. The bioaccessible Fe content also showed a pronounced improvement, with levels rising threefold from 0.21 mg/100 g in CH-GC to 0.59 mg/100 g in SF-CH-Fe 100.

KK exhibited a comparable pattern, as total iron increased significantly from 2.3 mg/100 g in KK-GC to 3.2 mg/100 g in SF-KK-Fe 100. Bioaccessible Fe also saw a substantial rise from 0.26 mg/100 g in KK-GC to 0.46 mg/100 g in SF-KK-Fe 100. These gains were dose-dependent, with greater fortificant additions leading to more pronounced improvements in both total and bioaccessible iron.

In contrast, Fe fortification was found to negatively impact Zn content in both the varieties studied (Figure 2c,d). For CH, total Zn dropped from 2.8 mg/100 g in GC to 2 mg/100 g in SF-CH-Fe 100. The bioaccessible Zn levels decreased in parallel from 0.26 in GC to 0.11 mg/100 g in SF-CH-Fe 100.

KK followed a similar trend: total and bioaccessible Zn declined from 1.8 mg/100 g to 1.7 mg/100 g, and from 0.25 to 0.10 mg/100 g in SF-KK-Fe 100 compared to KK-GC.

### 3.3. Single Fortification with Zinc—Effect on Total and Bioaccessible Zinc and Iron Content

**Figure 3:**
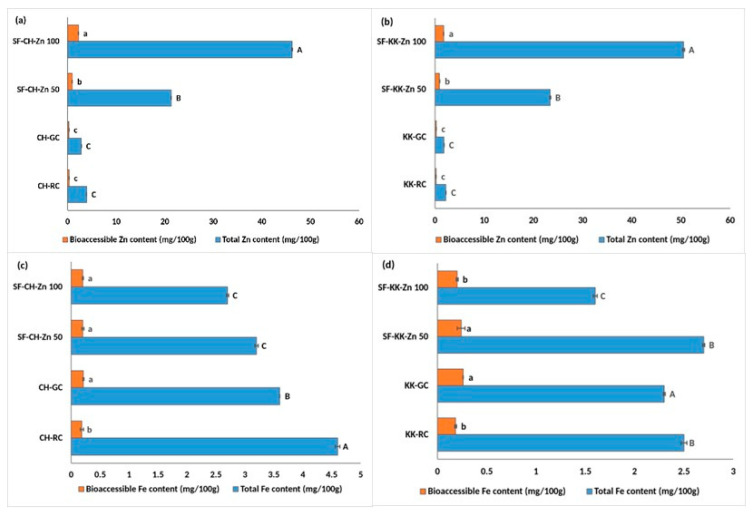
Effect of single fortification with ZnCl_2_ on the total and bioaccessible zinc and iron content in raw, germinated, and fortified Chennangi and Karungkuruvai rice samples. (**a**) Total and bioaccessible zinc content in Chennangi samples; (**b**) Total and bioaccessible zinc content in Karungkuruvai samples; (**c**) Total and bioaccessible iron content in Chennangi Samples; (**d**) Total and bioaccessible iron content in Karugkuruvai samples.Different lowercase letters (a–c) indicate significant differences (*p* < 0.05) in bioaccessible content among treatments within the CH and KK sample sets. Different uppercase letters (A–C) indicate significant differences (*p* < 0.05) in total content among treatments within the CH and KK sample sets. Data are presented as mean ± SD of three independent determinations; different alphabets indicate significant differences at *p* ≤ 0.05. (Note: CH—Chennangi, KK—Karungkuruvai; the suffixes R—raw/unprocessed, G—control/germinated, SF—Zn (50/100)—zinc fortified counterparts of different concentrations expressed in mg/L).

Bioprocess-based single fortification with zinc effectively increased both total Zn content and its bioaccessibility in the two varieties studied (Figure 3a,b). In CH, total Zn increased markedly from 2.8 in CH-GC to 46.2 mg/100 g in SF-CH-Zn 100. This was accompanied by a significant increase in bioaccessible Zn content from 0.26 in CH-GC to 2.2 mg/100 g in SF-CH-Zn 100. KK exhibited similarly pronounced enhancements, with total Zn increasing from 1.8 to 50.5 mg/100 g and bioaccessible Zn improving from 0.25 to 1.76 mg/100 g in KK-SF-Zn 100.

As expected, Zn fortification adversely affected iron concentrations (Figure 3c). In CH, total Fe declined significantly from 3.6 in CH-GC to 2.7 mg/100 g in SF-CH-Zn 100, with bioaccessible Fe showing an insignificant decrease from 0.21 in CH-GC to 0.20 mg/100 g in SF-CH-Zn 100.

A parallel pattern was observed in KK, where total Fe and bioaccessible Fe significantly decreased from 2.3 and 0.26 mg/100 g in KK-GC, to 1.6 and 0.20 mg/100 g, respectively, in SF-KK-Zn 100 (Figure 3d).

### 3.4. Double Fortification with Iron and Zinc—Effect on Total and Bioaccessible Iron and Zinc Content

The results of bioprocess-based double fortification are depicted in graphs where they are compared with the respective RC, GC, SF-Fe 100, and SF-Zn 50 for a better understanding of the effect of DF over the above treatments.

**Figure 4:**
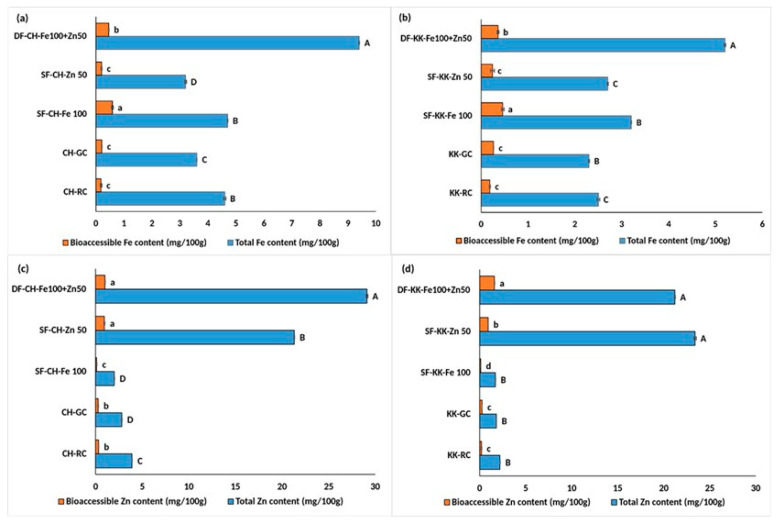
Effect of double fortification with NaFeEDTA and ZnCl_2_ on the total and bioaccessible iron and zinc content in raw, germinated, and fortified Chennangi and Karungkuruvai rice samples. (**a**) Total and bioaccessible iron content in Chennangi samples; (**b**) Total and bioaccessible iron content in Karungkuruvai samples; (**c**) Total and bioaccessible zinc content in Chennangi Samples; (**d**) Total and bioaccessible zinc content in Karugkuruvai samples. Different lowercase letters (a–d) indicate significant differences (*p* < 0.05) in bioaccessible content among treatments within the CH and KK sample sets. Different uppercase letters (A–D) indicate significant differences (*p* < 0.05) in total content among treatments within the CH and KK sample sets. Data are presented as mean ± SD of three independent determinations; different alphabets indicate significant differences at *p* ≤ 0.05. (Note: CH—Chennangi, KK—Karungkuruvai; the suffixes R—raw/unprocessed, G—control/germinated, DF—Fe100 + Zn50—double fortified counterparts expressed in mg/L).

In CH (Figure 4a), total Fe increased substantially from 3.6 in CH-GC and 4.7 in SF-CH-Fe 100 to 9.4 mg/100 g in DF-CH. While bioaccessible Fe rose approximately 2 times, from 0.21 in CH-GC to significantly higher levels of 0.46 mg/100 g in DF-CH. This was, however, significantly lower than the SF-CH-Fe 100.

Zn content showed remarkable enhancement in DF samples. In CH samples, total Zn increased from 2.8 in CH-GC and from 21.3 in SF-CH-Zn 50 to a significantly higher content of 29.1 mg/100 g in DF-CH (Figure 4b). The bioaccessible Zn improved significantly from 0.26 in CH-GC to 1 mg/100 g in DF-CH. This was also higher than the value (0.94) recorded in SF-CH-Zn 50.

In KK also (Figure 4c), total Fe increased significantly, from 2.3 in KK-GC to 3.2 in SF-KK-FE 100 to 5.2 mg/100 g in DF-KK. This was accompanied by a substantial rise (*p* < 0.05) in bioaccessible Fe from 0.26 in KK-GC to 0.36 mg/100 g in DF-KK. This was, however, significantly lower than 0.46 mg/100 g in SF-KK-Fe 100.

Zinc content in KK followed a similar pattern, with total Zn increasing (*p* < 0.05) from 1.8 in KK-GC to 21.2 mg/100 g in DF-KK and bioaccessible Zn improving from 0.25 in KK_GC to 0.92 in SF-KK-Zn 50 to 1.6 mg/100 g in DF-KK.

Thus, the DF carried out with Fe and Zn fortificants at 100 mg/L and 50 mg/L, respectively, yielded the most balanced and significant improvement in both Fe and Zn content and bioaccessibility, especially compared to untreated (RC) as well as the germination control.

### 3.5. Effect of Double Fortification on Total Phytate

A highly significant reduction in total phytate content was observed in the double-fortified HRVs compared with their raw/untreated (RC) counterparts (*p* < 0.001). In CH, phytate levels decreased from 2.4 ± 0.03 to 1.8 ± 0.04, whereas in KK, the levels declined from 2.2 ± 0.00 to 2.02 ± 0.02. The extent of reduction was greater in CH than in KK.

### 3.6. Cooking Characteristics of Double Fortified HRVs

#### 3.6.1. Length, Breadth, and Thickness

The above data were obtained for both uncooked and cooked samples. The results indicated that bioprocess-based fortification had a minimal influence on the physical dimensions of rice samples within both categories (Table 1). For example, the length of CH-RC was 5.21 mm, which reduced insignificantly to 4.90 mm in the CH-DF samples. The KK-RC had a greater length of 5.55 mm, while the fortified KK-DF showed a slight increase in length (5.92 mm). Breadth and thickness remained relatively consistent across both RC and fortified samples, with a slight decrease in the values. A similar trend was observed with these grain characteristics among the cooked raw/untreated and double fortified HRVs.

#### 3.6.2. Water Uptake Ratio

The water uptake ratios on cooking of double fortified HRVs were consistently higher than those of their raw/untreated counterparts. The CH-RC exhibited a water uptake ratio of 2.47, whereas the fortified sample showed an increased ratio of 2.61. Notably, KK demonstrated a substantial increase in the ratio from 1.86 in the raw sample to 3.33 in the bioprocessed fortified variant. These findings indicate that bioprocessed samples show a significantly higher water uptake ratio, a key parameter that positively influences the cooking quality of rice.

#### 3.6.3. Ratio of Kernel Elongation

The ratio of kernel elongation after cooking was as follows: CH-RC—0.23; CH-DF—0.24; KK-RC—0.15; KK-DF—0.1. A better kernel elongation ratio was observed in CH. In the case of KK, even though the lengths of cooked RC (6.38 mm) and DF (6.49 mm) samples were only marginally different, and on cooking, it was not reflected in the ratio due to the difference in the proportion of elongation.

#### 3.6.4. Cooking Time

**Figure 5:**
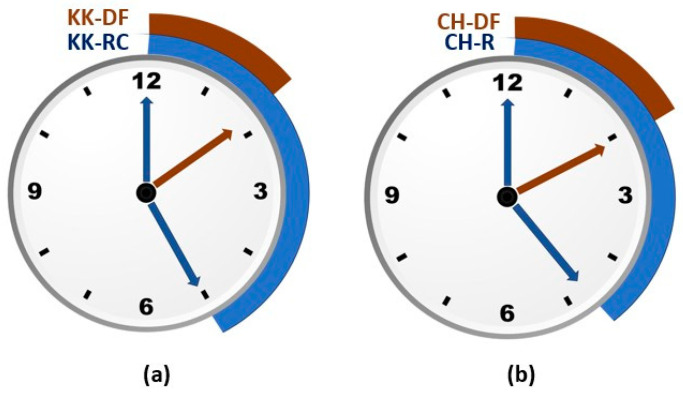
(**a**,**b**). Cooking time of raw/unprocessed control (RC) and double fortified (DF) Karungkuruvai and Chennangi.

Results demonstrate a substantial reduction in cooking time in bioprocessed double fortified rice varieties. In CH, a 12.35 min (22.57 to 10.22 min) reduction was observed (Figure 5a), while the effect was more pronounced in KK with a 17 min (25.10 to 8.16 min) reduction in cooking time (Figure 5b).

### 3.7. Sensory Quality of Double Fortified HRVs

**Figure 6:**
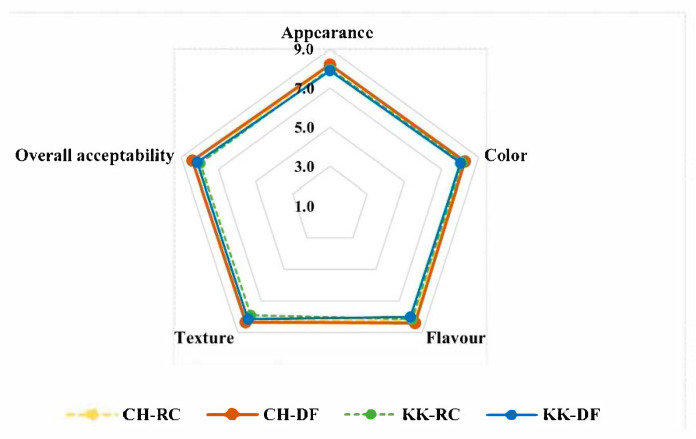
Sensory acceptability of raw/unfortified (RC) and double fortified (DF) Chennangi and Karungkuruvai.

Sensory evaluation data (Figure 6) from 24 adult panelists revealed that the double fortified versions of CH and KK were highly acceptable, similar to the untreated ones. The scores ranged between 7.9 and 8.2 for control, and between 7.9 and 8.4 for the double fortified samples, on a 9-point hedonic scale. Interestingly, the DF samples (8.2–8.4) had slightly better overall acceptability compared to their raw/untreated counterparts (8–8.1). There was no significant difference in appearance or color between the RC and DF HRVs. Notably, CH-DF recorded better scores in flavor (8.5) and texture (8.3) compared to CH-RC. The overall acceptability of the HRVs was in the order CH-DF > KK-DF > CH-RC > KK-RC.

In the triangle test, 85% of the panelists could identify CH-DF as the odd one. Similarly, in the case of KK, 90% of the panelists identified KK-DF as the odd one. Panelists attributed the differences perceived in the double fortified samples to better texture and flavor.

Preference tests revealed the following order among the samples studied: CH-DF > KK-DF > KK-RC > CH-RC. CH-DF was preferred by 53% of panelists, followed by KK-DF with 30%, while KK-RC and CH-RC were preferred by 10% and 7%, respectively, demonstrating a significant improvement in preference.

### 3.8. Dietary Iron and Zinc Contribution from Double Fortified HRVs

The DFHR (Double fortified heritage rice) of both Chennangi and KarungKuruvai was estimated to substantially contribute towards the daily requirements of iron and zinc for all the age groups (Table 2). The estimated daily intake (EDI) ranged between 1.3 and 2.3 mg for iron and 5 to 7 mg for zinc, even with a minimum consumption quantity (25 g). This contributes to substantial zinc adequacy (48–66% for adults) while iron contribution was modest (7–13% for adults). However, compared to adults, young children receive higher adequacy percentages (19–34% Fe and 100% Zn). An increase in consumption proportionately improves the iron and zinc adequacy across all demographics. Considering 100 g/day consumption (EDI: 5–9 mg of Fe and 21–29 mg of Zn), all the groups attained zinc adequacy, while iron adequacy ranged between 19 and 35% for pregnant and lactating women; 30–52% for adults and children above 4 years of age. A complete adequacy is obtained for both iron and zinc with consumption above 125 g/day across all age groups, while pregnant and lactating women need 175 g/day to obtain a high iron adequacy (34–61 mg) and complete zinc adequacy.

## 4. Discussion

### 4.1. Effect of Single and Double Fortification on Iron and Zinc Content of the HRVs

The iron content of the raw/untreated HRVs selected for the present study was found to be higher than a few pigmented rice cultivars [36,37] and similar to the one reported by Pathak et al. [38]; zinc content was comparable to other pigmented rice varieties [36,37,38]. Both iron and zinc contents of the RC-HRVs were found to be higher than those of various traditional rice genotypes [39] and even the improved rice varieties studied by Ayeleke et al. [40].

A few notable trends were observed during the process of single and double fortification of HRVs (Table 3). The germination process brought about a slight reduction in the total iron and zinc content, yet it improved their bioaccessibility. Single fortification with iron resulted in a remarkable increase in the total and bioaccessible content of iron. Similarly, upon fortifying with zinc, a pronounced increase in total and bioaccessible zinc was recorded. Interestingly, it was observed that HRVs had the tendency to accumulate significantly higher quantities of zinc. However, a significant and proportionate reduction in the competing element, i.e., reduction in iron upon fortifying with zinc and vice versa, was noted in both the HRVs. This may reflect mineral displacement or altered matrix interactions [41]. The worked-out ratio used for double fortification was also found to significantly improve the total iron and zinc contents. DF also enhanced the bioaccessible iron and zinc contents of the HRVs to a greater extent. Similarly to SF, DF samples also showed a trend of higher zinc uptake. Between the samples, CH showed better uptake of both iron and zinc compared to KK. Even though the initial iron and zinc content of CH was high, it could accumulate iron and zinc better than KK. This could be linked to the inherent genetic differences between the varieties. Understanding varietal differences in mineral uptake and storage can inform targeted selection for biofortification initiatives, potentially enhancing nutrient quality of crops and supporting public health efforts.

Iron fortification studies in brown rice, such as that by Wei et al. [25], reported comparable iron contents in unfortified and germinated samples, with significant increases only observed after direct iron fortification during germination. In the current study, CH demonstrated a notably higher iron uptake after fortification than those reported. The double-fortified HRVs (CH and KK) exhibited higher total iron and zinc contents than biofortified rice varieties, which typically reach about 1.5 mg/100 g for iron and 4.6 mg/100 g for zinc, as reported by Meenakshi et al. [42]. Furthermore, the bioaccessible fractions of iron and zinc in the experimental samples were higher than those observed in biofortified crops, indicating improved nutritional potential. Germination induces phytase activity, which degrades phytates and reduces their mineral-binding capacity [43,44], which is reflected in the improved bioaccessible content of iron and zinc, supported by the significant reduction in total phytate content upon germination.

When compared to fortified rice kernels (FRKs) commonly used in large-scale rice fortification programs, which generally contain 2 to 8 mg iron per 100 g, both CH and KK demonstrated similar or higher total iron content (5.2–9.4 mg/100 g). The bioaccessible iron content in these samples (0.5 mg/100 g) was also on par with values reported for extruded FRKs by Jyrwa et al. [45]. A clinical study by Pinkaew et al. [46] showed that the inclusion of extruded rice grains fortified with iron, zinc, and vitamin A (Fe: 20 mg/100 g; Zn: 18 mg/100 g) in school meals improved the zinc status of Thai school children. While the iron content in the experimental samples was lower, notably, the zinc content in both CH and KK was higher than the extruded fortified rice.

These findings suggest that co-fortification strategies can mitigate the antagonistic effects observed with individual mineral fortification by optimizing mineral interactions and improving overall bioavailability. Overall, the bioprocess-based fortification approach used for CH and KK not only matches or exceeds the mineral content and bioaccessibility of other fortified and biofortified rice products but also offers a natural and potentially more sustainable method for improving dietary iron and zinc intake.

### 4.2. Length, Breadth, and Thickness

The fortified rice samples (KK-DF and CH-DF) show a general reduction in these dimensions compared to their unfortified counterparts (KK-R and CH-R). However, when cooked, both fortified and unfortified samples exhibit an increase in size, with fortified cooked samples (KK-DFck and CH-DF-ck) showing a more pronounced increase. While fortification initially reduces the physical dimensions of rice, cooking appears to reverse this effect, likely due to water absorption and expansion. This aligns with findings that fortification may slightly alter physical properties but does not necessarily affect consumer acceptability negatively, as cooking restores desirable characteristics [47,48].

### 4.3. Water Uptake Ratio

The water uptake ratios of the rice samples CH and KK reveal significant differences between their unprocessed and double fortified forms. The data indicate that both rice varieties exhibit varying absorption characteristics, influenced by the fortification method and possibly their genetic traits. Reference [49] observed improvement in water absorption after 2–4 days of germination in brown rice. A study conducted by Choi et al. [50] on nineteen japonica and Tongil-type varieties revealed that varietal differences play a crucial role in their water uptake behavior. The study also observed that harder rice varieties (<12% moisture) have better water uptake capacity, which could also be a reason for the good uptake demonstrated by the experimental samples. Moisture content of the HRVs (RCHE: 5.79 ± 0.24%, RKKU: 6.87 ± 0.07%) was analyzed in our lab (unpublished data). Previous studies have also reported a negative correlation between grain moisture and hardness [51,52].

### 4.4. Ratio of Kernel Elongation

Kernel elongation is a desirable trait preferred by consumers [53], which is culturally rooted, driven by culinary application. Sood et al. [26] observed that the shape and arrangement of cells in the endosperm greatly determine the kernel elongation, apart from water uptake. The differences in the kernel elongation among rice varieties are a genetic trait, as shown by Sarmah et al. [54], who found that kernel length after cooking had high heritability estimates. Sima Chanu et al. [55] also had similar observations where a significant difference was not found in kernel elongation ratio after fortification.

### 4.5. Cooking Time

A 2.3 to 3-fold reduction in cooking time makes it convenient for consumers for quicker preparation of meals, while offering a healthier alternative to commonly consumed polished rice. Fortification carried out using the parboiling method in Sambha masuri (BPT-5204) rice had a longer cooking time (27–32 min) as reported by Chanu et al. [55]. Jiamyangyuen and Ooraikul [56] observed a 1.5-fold reduction in cooking time in germinated brown rice. A higher reduction in cooking time in CH and KK could be attributed to a longer germination time. Germination enhances the activity of amylase, which degrades starch and thereby causes a reduction in grain crystallinity [57,58]. The reduced hardness and improved water absorption capacity contribute to a notable reduction in cooking time.

### 4.6. Sensory Acceptability

The overall sensory acceptability of rice varieties depends on various factors. Varietal differences that influence flavor, texture [59], and intrinsic grain characteristics play a vital role. After selection of suitable rice cultivars, the cooking method used [60] and optimized water-to-rice ratio are important to obtain the desired texture [61]. Preparation of whole rice as part of mixed dishes allows better acceptance among consumers [62]. Choi and Seo [63] observed that demographics have a significant influence on textural perception and liking of foods. A standardized Indian dish—tomato bath used in the current study complemented the flavor profile of CH and KK, aiding in better acceptability. Utilization of germination as a bioprocess for fortification has led to further improvement in consumer preference and acceptability. Germinated brown rice has shown better cooking and textural properties [64]. It was also shown to have higher acceptability when incorporated in a functional beverage [65], indicating utility in various functional products. Furthermore, the results confirmed that the concentration employed for double fortification did not adversely affect texture or flavor, and preserved the positive impact of germination. Even though the difference was perceivable between the raw/untreated and double fortified HRVs in the triangle test, it was a favorable indicator of better acceptability among consumers. It was also reflected by the higher scores of double fortified HRVs in sensory attributes when compared to the raw/untreated HRVs.

### 4.7. Dietary Iron and Zinc Contribution

Rice is the primary staple for more than half the world’s population. Countries like the USA have minimal rice consumption, whereas Asian and African countries have a high daily consumption [66]. The quantity of rice consumed also varies with the age group of the population. A comprehensive range (25–175 g) considered in the current study provided a systematic understanding of the potential dietary contribution of iron and zinc towards various age groups of the population. According to the USFDA [67], a food item is considered a good source of a particular nutrient when the item contributes between 10 and 20% of the daily requirements of the nutrient. Similarly, a food item is categorized as an excellent source when the contribution is >20% and a high potency source when it completely meets the daily requirement (100%). The DFPRs have been shown to be a good to excellent source of iron and an excellent to high potency source of zinc for children and adults, even with a minimum consumption of 25 g/day.

## 5. Conclusions

The bioprocess-based fortification approach studied offers a practical and scalable solution to produce quick-cooking, double-fortified pigmented rice with enhanced nutritional and functional benefits and cooking characteristics. It leverages natural biochemical processes to improve both micronutrient concentration and bioaccessibility, while preserving beneficial secondary metabolites. Compared to genetically modified rice varieties, this method preserves the natural food matrix, maintaining the intrinsic health benefits of the grain while avoiding regulatory and consumer acceptance hurdles associated with genetic modification. Additionally, fortified pigmented rice produced through bioprocessing is more wholesome than rice analogs or synthetic supplements, providing a comprehensive nutrient profile that supports overall health. It is a low-tech, cost-effective process that makes it particularly suitable for decentralized production systems, facilitating wider adoption and accessibility. This approach holds good promise for addressing micronutrient deficiencies in vulnerable populations, particularly in low-resource settings where rice is a staple food. It has the potential to strengthen nutrition security in the population and directly supports the Sustainable Development Goals (SDG) 2.2—to end all forms of malnutrition—and SDG 3—promoting good health and well-being. Further research is underway to refine the fortification process and enhance its effectiveness and nutritional impact. In addition, fortification studies are being extended to other light-bran heritage and pigmented rice varieties, thereby broadening the applicability of this methodology and enhancing its contribution to nutrition security.

## Figures and Tables

**Table 1:** Length, breadth, and thickness of raw/untreated (RC) and double fortified DF-HRVs.

S.No.	Sample	Length (mm)	Breadth (mm)	Thickness (mm)
1	CH-RC	5.21 ^ab^ ± 0.15	2.66 ^a^ ± 0.16	1.87 ^ab^ ± 0.13
2	CH-DF	4.90 ^a^ ± 0.23	2.55 ^a^ ± 0.17	1.71 ^a^ ± 0.11
3	KK-RC	5.55 ^abc^ ± 0.27	2.68 ^ab^ ± 0.18	2.07 ^abcd^ ± 0.12
4	KK-DF	5.92 ^bcd^ ± 0.13	2.58 ^a^ ± 0.19	1.92 ^abc^ ± 0.14
5	CH-RCck	6.40 ^d^ ± 0.44	2.87 ^ab^ ± 0.27	2.27 ^cd^ ± 0.14
6	CH-DFck	6.06 ^cd^ ± 0.33	2.99 ^ab^ ± 0.21	2.20 ^bcd^ ± 0.17
7	KK-RCck	6.38 ^cd^ ± 0.36	3.24 ^b^ ± 0.27	2.27 ^cd^ ± 0.16
8	KK-DFck	6.49 ^d^ ± 0.31	3.08 ^ab^ ± 0.15	2.33 ^d^ ± 0.12

Data are presented as mean ± SD of three independent determinations; different alphabets indicate significant differences at *p* ≤ 0.05. (Note: CH—Chennangi, KK—Karungkuruvai; the suffixes R—raw/unprocessed; DF—double fortified, ck—respective cooked counterparts).

**Table 2:** Estimated dietary contribution of double fortified HRVs towards the daily iron and zinc requirements of different age groups.

Age (yrs.)	Children 1 to 3 Y		Children (≥4 Y) and Adults		Pregnant and Lactating Women	
DFHR (g/Day)	Fe	Zn	Fe	Zn	Fe	Zn
	Percent (%) Contribution Towards RDI					
25	19–34	100	7–13	48–66	5–9	41–56
50	37–67	100	14–26	96–100	10–17	82–100
75	56–100	100	22–39	100	14–26	100
100	74–100	100	29–52	100	19–35	100
125	93–100	100	36–65	100	24–44	100
150	100	100	43–78	100	29–52	100
175	100	100	51–91	100	34–61	100

DFHR—Double fortified-heritage rice (CH and KK); Fe—Iron; Zn—Zinc.

**Table 3:** Summary of the effect of single and double fortification on the iron and zinc content (total and bioaccessible) in heritage rice varieties.

Group	Variety	Iron Content		Zinc Content	
		Total	Bioaccessible	Total	Bioaccessible
Control-Germination	CH	↓	↑	↓	↓
	KK	↑	↑	↓	↑
SF-Iron fortification	CH	↔	↑	↓	↓
	KK	↑	↑	↓	↓
SF-Zinc fortification	CH	↓	↔	↑↑	↑↑
	KK	↓	↔	↑↑	↑↑
DF-Double fortification	CH	↑↑	↑↑	↑↑	↑↑
	KK	↑↑	↑↑	↑↑	↑↑

Note: Red arrows indicate a decrease in iron or zinc content, while green arrows indicate an increase. Single arrows represent a modest change, and double arrows represent a significant change. Left-right arrows indicate no observable difference in iron or zinc content.

## Data Availability

The original contributions presented in the study are included in the article, further inquiries can be directed to the corresponding author.
